# Historical and Cross-Country Differences in Life Satisfaction Across Retirement in Germany and Switzerland From 2000 to 2019

**DOI:** 10.1093/geronb/gbad066

**Published:** 2023-06-09

**Authors:** Georg Henning, Isabel Baumann, Oliver Huxhold

**Affiliations:** German Centre of Gerontology (DZA), Berlin, Germany; Institute of Public Health, ZHAW Zurich University of Applied Sciences, Winterthur, Switzerland; Centre for the Interdisciplinary Study of Gerontology and Vulnerability (CIGEV) and National Centre of Competence in Research “Overcoming Vulnerability: Life Course Perspectives” (NCCR LIVES), University of Geneva, Switzerland; German Centre of Gerontology (DZA), Berlin, Germany

**Keywords:** Education, Life satisfaction, Retirement, Social change

## Abstract

**Objectives:**

Recent trends, such as changes in pension systems or cohort differences in individual resources, have altered the face of retirement transitions. Little is known about how these trends have affected older people’s life satisfaction around retirement age in the past decades. In this study, we investigated how levels and changes in life satisfaction before and after retirement changed over historical time in Germany and Switzerland.

**Methods:**

We used longitudinal data from the German Socioeconomic Panel Study and the Swiss Household Panel (SHP) from 2000 to 2019. Level, preretirement change, and short- and long-term change in life satisfaction (0–10) after retirement were predicted by year of retirement (2001–2019) in a multigroup piecewise growth curve model.

**Results:**

We found improvements in levels of life satisfaction and preretirement changes in life satisfaction with historical time in both countries. Furthermore, we found that unlike in Switzerland, short-time changes in life satisfaction across retirement improved over historical time in Germany.

**Discussion:**

Our findings imply that life satisfaction trajectories around retirement age have improved over the last 20 years. These findings may be explained by general improvements in the health and psychosocial functioning of older people. More research is needed to show for whom these improvements are stronger or weaker and if they will be maintained in a changing retirement landscape.

In many OECD countries, macro-level conditions for retirement have changed over the last few decades (OECD, 2019). For instance, statutory retirement ages have been raised or rendered flexible in many countries (Hofäcker et al., 2016). Macro-level factors are assumed to affect resource levels and those, in turn, affect retirement adjustment (Wang et al., 2011). If these factors differ between retirees at different historical time points, their resource levels and, consequently, their adjustment to and their well-being in retirement will differ as well (Henning, Johansson, et al., 2022; Henning, Segel-Karpas, et al., 2022).

In the current study, we first investigate if life satisfaction across retirement has changed between 2000 and 2019; then, we determine if differences exist between Germany and Switzerland, which share similar institutional contexts but have undertaken different changes in policy in recent years. Research on historical differences is particularly important in the context of the dramatic changes in retirement policies that have taken place in many Western countries over the first two decades of the 21st century. Especially for social scientists, such research is important for understanding how pension regimes and public views on aging affect older adults’ experiences in retirement (Henning, Johansson, et al., 2022) and how older workers react to changes in retirement policies (Henkens et al., 2018). Apart from one study (Richardson et al., 2019), there is also very little research comparing retirement adjustment across countries. A comparison of different countries may elucidate the role of macro-societal factors in individual adjustments to retirement and improve our knowledge of the universality of changes in well-being centered around retirement.

## Life Satisfaction Across Retirement—Dynamic Change and Interindividual Differences

Retirement marks the beginning of a new life phase and the need for adaptation. Challenges in the transition to retirement include dealing with the loss of work-related resources such as income, a daily structure, and social contacts (Luhmann et al., 2012), as well as developing a satisfactory retirement routine (Wetzel et al., 2016). Opportunities in retirement include increased autonomy, stress reduction, and more free time (Luhmann et al., 2012; Stenling et al., 2021). Life satisfaction is a common measure indexing the success of adjusting to changes in living conditions (Hansson et al., 2017).

Retirement adjustment is a dynamic process (Atchley, 1976; Zhan et al., 2022). In a meta-analysis, Luhmann et al. (2012) found a small decline in cognitive-evaluative well-being (e.g., life satisfaction) after retirement and adaptation later on but concluded that retirement is a rather neutral event. However, more recent studies with more complex designs identified short-term improvements in life satisfaction and other measures of well-being after retirement (Åhlin et al., 2020; Wetzel et al., 2016). This short-term increase has been seen as a “honeymoon” period of increased autonomy and relief from work (Atchley, 1976; Stenling et al., 2021). Nevertheless, life satisfaction often declines after the first years of retirement (Sohier et al., 2021; Wetzel et al., 2016), which may be related to functional decline (Pinquart & Schindler, 2007) or struggles to adjust to everyday challenges in retirement (Atchley, 1976).

To address the dynamic nature of this adjustment process, longitudinal assessments over several years are necessary. Here, we focus our analysis on retirement in Switzerland and Germany. Previous studies using the same data sets have shown that life satisfaction decreases before and after retirement (Wernli et al., 2015; Wetzel et al., 2016). In some German studies, retirees also exhibited short-term increases in life satisfaction after retirement (Henning, Segel-Karpas, et al., 2022; Weber & Hülür, 2020; Wetzel et al., 2016).

In both Switzerland and Germany, changes in life satisfaction after retirement seem to depend on gender, well-being facets, and working conditions (Ryser & Wernli, 2017), as well as preretirement work status (Schmälzle et al., 2019), which highlights the heterogeneity of the retirement experience. Previous studies have identified a multitude of predictors of well-being across retirement (for a review, see Henning et al., 2016), such as social contacts and health, as well as the individual job and transition characteristics (e.g., voluntary vs involuntary; gradual vs abrupt).

According to the interdisciplinary resource-based dynamic perspective on retirement adjustment (from hereon: resource perspective) by Wang et al. (2011), fluctuations and differences in retirement adjustments are explained by intra- and interindividual differences in the resource capacity of the retirees. Specifically, antecedents on the macro-level (e.g., norms, pension systems), organizational level, job level, household level, and individual level influence the availability of physical, cognitive, motivational, financial, social, and emotional resources. Retirees with more resources adjust easier than those with fewer resources, and when an individual’s resources fluctuate over time, retirement adjustment (and life satisfaction) will too. Social inequalities in resources thus go hand in hand with social inequalities in retirement adjustment (Henning, Johansson, et al., 2022; König et al., 2018; Wetzel et al., 2016).

### The Role of the Historical Context

Research on retirement and life satisfaction has typically focused on psychosocial predictors or the individual retirement context (Henning et al., 2016). We focused on contextual factors instead, namely, the role of historical time (Henning, Johansson, et al., 2022; Henning, Segel-Karpas, et al., 2022) and country (Richardson et al., 2019). We investigated typical trajectories of life satisfaction across retirement in Germany and Switzerland and studied historical changes and stabilities.

Following recent papers on the subject, we use the term “historical differences,” which refers to all “interindividual differences that are consequences of historical changes” (Drewelies et al., 2019, p. 1022) and aims at avoiding the distinction of period and cohort effects, which we do not disentangle in the current paper. Few studies have considered historical differences in life satisfaction across retirement (Henning, Johansson, et al., 2022). Based on the resource perspective, life satisfaction during a transition to retirement is dependent on resource capacity. However, resources may differ over historical time, and so should life satisfaction during the retirement transition. Potential reasons for systematic differences in resources over time include pensions and work reforms. Since the 1990s, many governments have implemented new laws aimed at increasing the work participation of older adults and raising the retirement age to lower the burden on public pension systems (Hofäcker et al., 2016).

In the present study, we focused on the historical period from 2000 to 2019. At this time, in Germany, the statutory retirement age has begun to gradually increase from 65 to 67. Opportunities for early retirement have been limited. In cases where this was still possible, it was usually accompanied by pension cuts (Hess, 2018). A number of exceptions allowing early retirement without larger pension cuts for particular groups (e.g., women or long-term unemployed workers) have also been abandoned (Brussig et al., 2016).

In Switzerland, men’s statutory retirement age has not changed since the introduction of the old-age pension system in 1948. However, women’s statutory retirement age was raised from 62 to 63 in 2001 and from 63 to 64 in 2005 (BSV, 1996).

### Historical Differences in Well-Being and Adjustment Across Retirement

Changes in macro-conditions are likely to lead to historical differences in resources and consequentially to differences in life satisfaction. Although previous studies showed secular improvements in the health and psychosocial resources of older adults (Drewelies et al., 2019), this may not be the case during the retirement transition: First, at least in Germany, pension income seems to decrease (Deutsche Rentenversicherung Bund, 2020), which may affect retirees’ adjustment negatively. Similarly, one Dutch study found a negative effect of a reduction in old-age pension benefits in the Netherlands on mental health among workers affected by the reform (Grip et al., 2012). Moreover, although retiring at later ages is often associated with improved well-being (Pinquart & Schindler, 2007), increases in the statutory retirement age may actually decrease the adjustment potential of later-born cohorts. Agency in deciding about one’s retirement age seems to be crucial for a happy retirement transition (Hershey & Henkens, 2014). For example, only those older workers who are able to work until statutory retirement age tend to show high life satisfaction (Baumann et al., 2022).

So far, there have been few studies on historical differences in retirement adjustment. To our knowledge, no such study has been done using Swiss data. Concerning Germany, Henning, Johansson, et al. (2022) used data from the German Ageing Survey (DEAS) to compare four groups of retirees (1996, 2002, 2008, and 2014) and found that retirement satisfaction seemingly increased between 1996 and 2008, although other measures of adjustment remained stable. Using the same data set, Henning, Segel-Karpas, et al. (2022) found that levels and changes in life satisfaction were comparable among three groups of individuals, either retiring between the survey waves of 1996 and 2002, between the waves of 2002 and 2008, or between the waves of 2008 and 2014. However, levels of positive affect seemed to have improved with historical time, whereas increases in positive affect across the transition were diminished in later samples. Taken together, some domain-specific historical differences in retirement adjustment seem to exist in Germany between 1996 and 2014.

These results do not imply large historical differences in life satisfaction across retirement. Nevertheless, we assume that distinguishing between short- and long-term retirement changes and the inclusion of more recent data may paint a different picture. Short-term changes in life satisfaction may be more prone to historical effects, as changes in the conditions of the retirement transition may mainly affect feelings of agency and autonomy early on in retirement (cf. Henning, Segel-Karpas, et al., 2022)—later on in their retirement, individuals are likely to adapt. Furthermore, both studies mentioned previously only examined the period of 1996 to 2014, and the effects of the raised statutory retirement age could be more pronounced in later years. Hence, we assume:

*H1*: Short-term increases in life satisfaction across retirement decreased with historical time.

### Cross-Country Differences and Social Inequalities

Comparing different countries can bring additional insights into the study of retirement adjustment: A recent study showed that changes in the quality of life following late-life work exits differed between European countries, with welfare state type explaining 62% of the between-country variance (Richardson et al., 2019). German and Swiss pension regimes have three-pillar systems (OECD, 2019) that feature public pensions, occupational pensions, and private savings. However, whereas occupational pensions are mandatory for most employees in Switzerland, they are voluntary in Germany. Moreover, the retirement age in Switzerland has been more stable than in Germany. Assessing the effect of different policy developments demands a comparative approach that allows simultaneous measurement of changes over time and differences in these changes between countries (Baumann & Madero-Cabib, 2021; Börsch-Supan et al., 2018). Given the reasoning outlined previously, we assume the following:

*H2*: The historical decrease in change in life satisfaction is smaller in Switzerland than in Germany.

Previous studies have shown that secular changes in well-being and health are usually less positive among individuals with lower social status and education (Infurna et al., 2021). Researchers have argued that historical developments may increase existing inequalities in retirement timing (Hofäcker et al., 2015). Lower-educated older workers especially are less likely to choose when to retire. Their financial resources do not allow them to accept the pension cuts associated with retiring early; at the same time, working longer is often not possible and may even be detrimental to their health (Hofäcker & Radl, 2016). Consequently, Henning, Johansson, et al. (2022) found that historical improvements in retirement satisfaction were mainly present among white-collar workers, not among blue-collar workers. Thus, we assume the following:

*H3*: The historical decrease in change in life satisfaction is larger for lower-educated retirees than for higher-educated retirees.

Women’s labor force attachment has substantially increased in recent decades. In younger cohorts of older workers, more women are employed and work more hours than in older cohorts of older workers (Turek et al., 2022). Furthermore, both countries have increased their retirement age for women. Therefore, we expect the following:

*H4*: The historical decrease in change in life satisfaction is larger among women than among men.

### How to Define Retirement

Traditionally, retirement has often been seen as a time in which individuals stop working and take out retirement pensions. However, retirement transitions have become more complex over the last decades, with many retirees still working to some extent during retirement (Engstler & Romeu Gordo, 2014) and others exiting the workforce early while receiving unemployment benefits or disability pensions (Schmälzle et al., 2019). Accordingly, definitions of retirement differ between studies, with some studies relying on self-reports and others on income source or work status (Eyjólfsdóttir et al., 2021). Nevertheless, in the context of Germany and Switzerland, public pensions are still central to retirement income, and the retirement transition is typically seen as the point in time at which people start to take out these pensions (Henning, Johansson, et al., 2022; Wetzel et al., 2016).

### The Present Study

In the present paper, we studied levels of short- and long-term changes in life satisfaction in Germany and Switzerland, and their historical development from 2000 to 2019. We further studied educational and gender differences in these historical developments. Finally, we determined whether the historical effects stayed the same if we considered the historical shift toward later retirement ages, as the main aim behind many pension reforms is to delay retirement (Hofäcker et al., 2016).

## Method

### Sample

Our analyses were based on the German Socioeconomic Panel (SOEP, Goebel et al., 2019) and the Swiss Household Panel (SHP, Tillmann et al., 2016). Both surveys are ongoing household panel surveys that are conducted annually. The SOEP started in 1984, and the SHP began in 1999. In the SHP, data on life satisfaction were available from 2000 onwards. We focused on the overlapping time period before the COVID-19 pandemic (2000–2019) because of the potential impact of the pandemic. We included individuals who retired within this period and whose retirement was legally possible (i.e., ≥58+ in Switzerland and ≥60 in Germany) and realistic (i.e., ≤67). We excluded unrepresentative subsamples in the SOEP. Additional information is reported in Supplementary Appendix B. To facilitate replicability, STATA files for sample generation can be found on the server of the Open Science Foundation (OSF; see Author Note 1).

The final sample consisted of *n* = 3,811 individuals from the SOEP and *n* = 1,806 individuals from the SHP. For every retiree, we used data from up to 3 years before their retirement, the retirement year, and up to 4 years after retirement. This time frame included both pre- and postretirement changes over several years and enabled reliable estimates of historical differences across transitions over 19 years. A table showing the number of observations available for each time point and the number of transitions that occurred in each year can be found in Supplementary Tables 1 and 2. Table 1 shows the descriptive statistics for age, gender, and education.

**Table 1.** Descriptive Statistics

	SOEP (*n* = 3,811)	SHP (*n* = 1,806)
Age at retirement	*M* (*SD*) = 63.22 (1.96)	*M* (*SD*) = 64.35 (1.34)
Gender	2,003 women 1,808 men	978 women 828 men
Education	405 with low education 3,186 with middle or high education	139 with low education 1,667 with middle or high education

*Notes*: SHP = Swiss Household Panel; SOEP = German Socioeconomic Panel Study.

### Measures

#### Retirement year

We defined retirement as beginning from the first receipt of retirement pensions. This definition was chosen because of the large role of public pensions in both systems and to ensure comparability over historical time and across surveys. For example, due to the survey structure, working retirees would have been excluded from the SHP but not from the SOEP. Both surveys included items on individual incomes and income types. We used the year of retirement as a measure of historical time. In the SOEP, participants were asked about their income in the last year. We included individuals who retired between 2001 and 2019.

#### Life satisfaction

In both samples, life satisfaction was assessed as a single item on an 11-point scale from 0 = *completely dissatisfied (SOEP)*/*not at all satisfied (SHP)* to 10 = *completely satisfied*.

#### Education

Both surveys included International Standard Classification of Education (ISCED-97) codes. There are six categories within the ISCED system. Due to the important role of vocational secondary education in German-speaking countries (Korpi & Mertens, 2003), we assumed that the main difference may be between those with less than secondary education and those with at least secondary education. Thereby, we distinguished between low (inadequate and general elementary) education and middle or high education (coded as low = 0 and middle or high = 1).

#### Gender

Gender was included as a dichotomous variable (0 = woman, 1 = man).

#### Age

Retirement age (58–67 years of age in Switzerland, and 60–67 years of age in Germany) was included as a covariate and assessed at the retirement year. In the models, age was centered around age 60, so adjusted intercepts of level and slopes can be interpreted as the mean level and change for a retiree aged 60 at the year of retirement.

### Analysis

**Figure 1.**
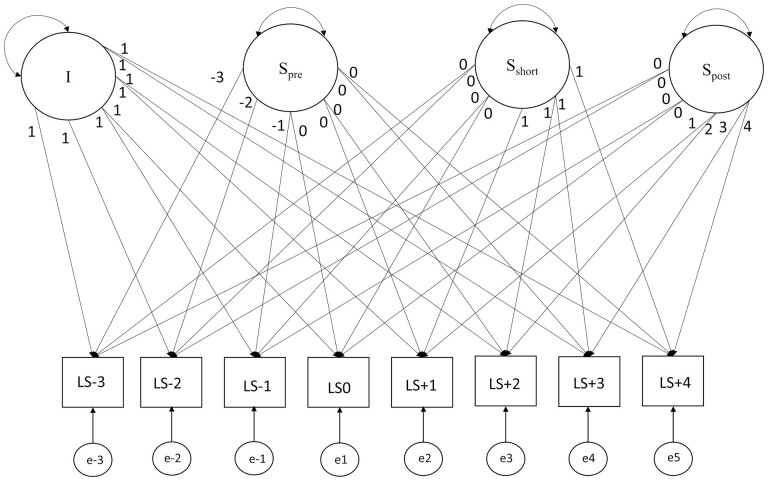
A piecewise latent growth model of change in life satisfaction from 3 years before retirement to 5 years in retirement.

All analyses were conducted using MPlus 8.4. Our baseline model was a multigroup piecewise latent growth curve model with country (Germany [SOEP] vs Switzerland [SHP]) as the grouping variable. This model is an extension of the basic latent growth curve, a two-factor structural equation model that consists of an intercept (i.e., the level of life satisfaction) and a slope parameter (i.e., the linear change over time). Previous research used a two-slope growth model with one slope before and one after retirement (Zulka et al., 2021). This extension allowed us to distinguish the rate, as well as predictors, of pre- and postretirement change. We built on these studies by modeling change in life satisfaction, including an intercept, set to the first year of retirement, a linear preretirement slope (from 3 years before retirement to the first year of retirement), and a linear postretirement slope (between the first year before and the fifth year in retirement). In addition, we added a short-term slope to account for potential short-term effects. This slope represents an additional change in the first year after retirement over and above the long-term postretirement change (cf. Wetzel et al., 2016). This allowed us to separately test predictors of life satisfaction levels before retirement, pre- and postretirement development, as well as additional short-term change directly after retirement. An illustration of the structural model can be found in Figure 1. The Mplus code for this model can be found on the OSF server (see Author Note 2).

As a first step, we investigated cross-country differences in life satisfaction by examining whether intercepts and slopes could be set to equality between countries without a significant loss in model fit (i.e., performing *χ*² difference tests).

To test Hypothesis 1, we added retirement years as a predictor of intercepts and slopes. To test Hypothesis 2, we tested whether the predictor effects could be set to equal across countries, again using a *χ*² test. To test Hypotheses 3 and 4, we added gender and education, as well as gender × retirement year and education × retirement year interaction effects. Again, we tested whether the main and interaction effects could be set to equal in both samples, using *χ*² tests. Finally, we repeated the analysis, controlling for the effects of retirement age.

The alpha level was set to 0.05. Missing data were handled using a full information maximum likelihood estimation with robust standard errors (FIML). FIML does not impute new values but adjusts parameter bias via a maximum likelihood function that uses missing values and covariation patterns in the data set as input (Enders, 2009). Model fit was evaluated with a combination of the comparative fit index (CFI), the Tucker–Lewis index (TLI), the root-mean-square error of approximation (RMSEA), and the standardized root-mean-square residual (SRMR). CFI and TLI values of 0.90 and higher, and SRMR and RMSEA values of 0.08 and lower, are considered an acceptable fit (Marsh, 2007).

## Results

### Changes Across Retirement

The unconditional growth curve indicated a very good fit to the data (CFI = 0.994, TLI = 0.992, SMR = 0.024, RMSEA = 0.022, 90% CI [0.017;0.028]; see Author Note 3). The results of all following χ² tests can be found in Supplementary Appendix C. The final model was a model with different intercepts and short-term changes but the same pre- and postchange in the German and Swiss samples. In both countries, life satisfaction did not change significantly before retirement (*M*_preretirement slope_ = −0.01, *SE* = 0.01, *p* = .151), or afterwards (*M*_postretirement slope_ = 0.01, *SE* = 0.01, *p* = .508). The SHP participants had a higher level of life satisfaction than the SOEP participants (*M*_SHP_ = 8.16, *SE* = 0.03 vs *M*_SOEP_ = 7.02, *SE* = 0.03). SOEP participants showed an increase in life satisfaction early after the transition (*M*_Short-term slope SOEP_ = 0.14, *SE* = 0.03, *p* < .001), which was not present in the SHP (*M*_Short-term slope SHP_ = 0.01, *SE* = 0.03, *p* = .809). An illustration of average changes in life satisfaction in both samples (Supplementary Figure 1), as well as all parameters of this model (Supplementary Table 3), can be found in the Supplementary Materials.

### Historical Effects

**Figure 2.**
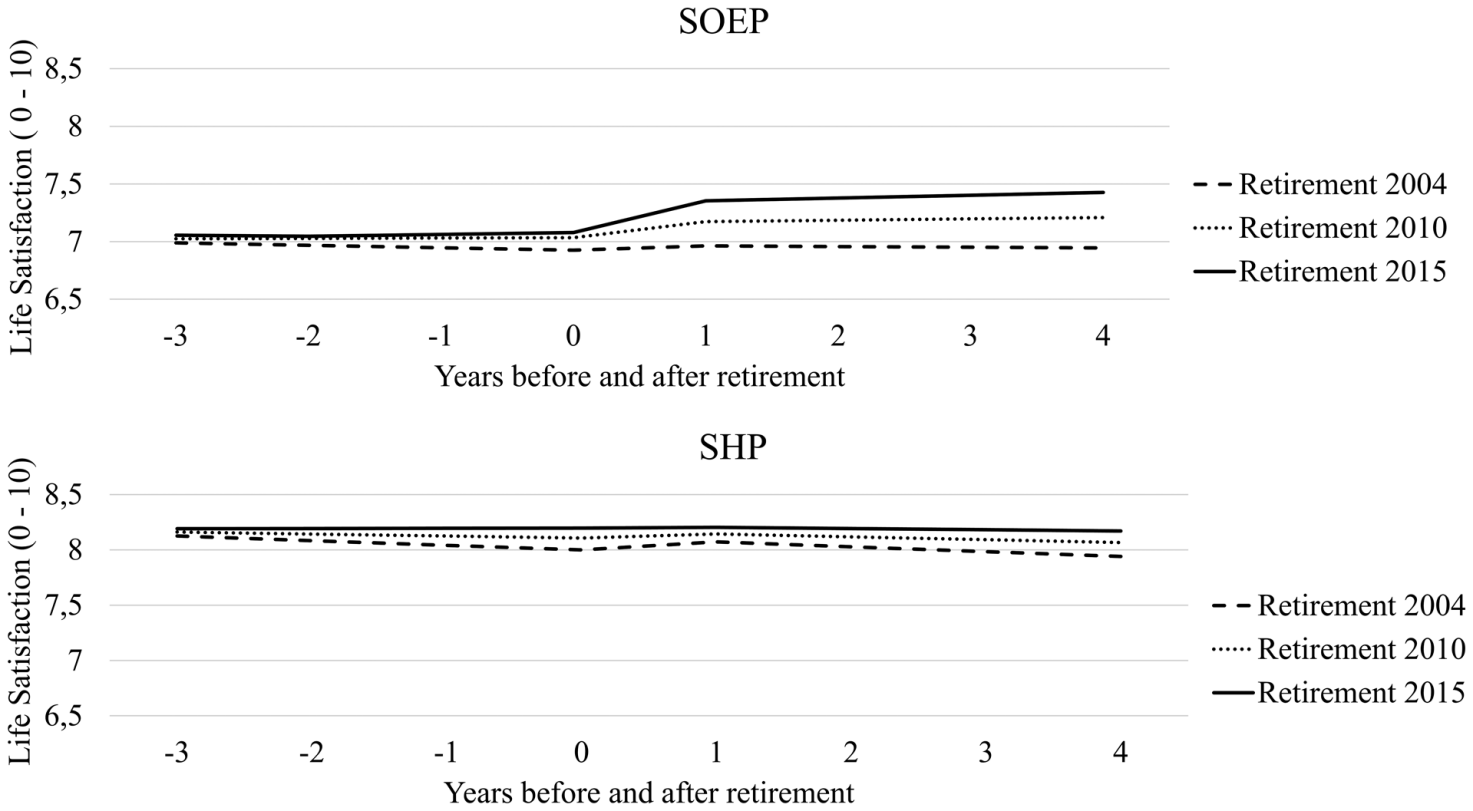
Historical differences in life satisfaction across retirement. Historical improvements are significant for intercepts, and preretirement slopes in both samples, but for the short-term change only in the SOEP.

After adding retirement year as a predictor, χ² tests led to a model with the same historical effects on preretirement level, preretirement change, and postretirement change in life satisfaction. However, the effects of short-term change differed between countries. The parameters of our final model can be found in Supplementary Table 4. Contrary to Hypotheses 1 and 2, short-term increases in life satisfaction became stronger with historical time in Germany (*B* = 0.01, *SE* = 0.01, *p* = .005), but not in Switzerland. Levels of life satisfaction (*B* = 0.01, *SE* = 0.00, *p* < .001) and preretirement change (*B* = 0.01, *SE* = 0.00, *p* = .003) grew more positive over time in both countries, but there were no significant historical differences in postretirement change. These effects are illustrated in Figure 2, which models trajectories for individuals who retired in 2004, 2010, and 2015. Panel A shows historical differences in Germany, while Panel B does the same for Switzerland.

### Historical Effects of Gender and Education

After controlling for the effects of gender and education, χ² tests led to the same models and the effects of retirement years remaining unchanged (see Table 2). In Switzerland, men had higher levels of life satisfaction than women (*B* = 0.15, *SE* = 0.06, *p* = .030), but not in Germany. Genders did not differ concerning change. Higher education was associated with a higher level of life satisfaction (*B* = 0.39, *SE* = 0.08, *p* < .001), but not with change.

**Table 2.** Predicting the Level and Change in Life Satisfaction in SOEP and SHP (Without Controlling for Age at Retirement)

	SOEP (*n* = 3,811)			
	Level, *B* (*SE*)	Preretirement slope, *B* (*SE*)	Short-term slope, *B* (*SE*)	Postretirement slope, *B* (*SE*)
Intercept	6.58 (0.09)***	−0.07 (0.03)*	0.05 (0.09)	−0.05 (0.03)
Retirement year (0 = 2001)	0.02 (0.00)***	0.004 (0.00)**	0.01 (0.01)**	0.00 (0.00)
Gender (0 = female, 1 = male)	−0.08 (0.05)	0.02 (0.01)	−0.06 (0.04)	0.02 (0.02)
Education (0 = low, 1 = middle and high)	0.39 (0.08)***	0.03 (0.03)	−0.02 (0.09)	0.03 (0.03)
Residual variance	1.99 (0.08)***	0.05 (0.01)****	0.43 (0.12)***	0.06 (0.02)***

	SHP (*n* = 1,806)			
	Level, *B* (SE)	Preretirement slope, *B* (*SE*)	Short-term slope, *B* (*SE*)	Postretirement slope, *B* (*SE*)
Intercept	7.55 (0.10)***	−0.09 (0.04)*	0.19 (0.11)	−0.09 (0.03)*
Retirement year (0 = 2001)	0.02 (0.00)***	0.004 (0.00)**	−0.01 (0.01)	0.00 (0.00)
Gender (0 = female, 1 = male)	0.15 (0.06)*	0.02 (0.01)	−0.06 (0.04)	0.02 (0.02)
Education (0 = low, 1 = middle and high)	0.39 (0.08)***	0.03 (0.03)	−0.02 (0.09)	0.03 (0.03)
Residual variance	1.36 (0.10)***	0.04 (0.01)**	0.12 (0.14)	0.04 (0.02)**

*Notes*: CFI = comparative fit index; CI = confidence interval; RMSEA = root-mean-square error of approximation; SHP = Swiss Household Panel; SOEP = German Socioeconomic Panel Study; SRMR = standardized root-mean-square residual; TLI = Tucker–Lewis index. Model fit: CFI = 0.994, TLI = 0.992, RMSEA = 0.018, 90% CI (0.014; 0.023); SRMR = 0.020.

*p < .05 ** *p* < .01 ***p < .001.

Next, we added interaction effects and repeated the *χ*² tests. Coefficients can be found in Supplementary Table 5. In the final model, all main effects could be set equal except for the main effect of education on levels of life satisfaction, which was significant in Switzerland (*B* = 0.74, *SE* = 0.22, *p* = .001) but not in Germany. All interaction effects, except for the education × retirement year interaction effect on short-term change, were set equal as well. However, all interaction effects were estimated as not significant. Thus, H3 (i.e., more positive historical developments among those with higher education) and H4 (i.e., larger historical differences among women) were not supported.

### The Role of Retirement Age

Finally, we added retirement age as a predictor, as later retirement transitions may be partly responsible for the effects we found. We repeated previous analyses separately with models with and without interaction effects. The analytical steps are detailed in the Supplementary Appendix C).

In the model without interaction effects (see Table 3), we found that higher retirement age was associated with a higher life satisfaction (*B* = 0.09, *SE* = 0.01, *p* < .001). People who retired at an older age were more satisfied but did not differ in terms of change after retirement from people who retired at a younger age. The association of retirement years with a level of life satisfaction before retirement was no longer significant after accounting for differences in retirement age. Historical effects on preretirement change as well as on short-term change in Germany, but not in Switzerland, remained significant, and the effect on postretirement change was now positive and significant as well. Including interaction effects did not provide additional insights (see Supplementary Table 6).

**Table 3.** Predicting the Level and Change in Life Satisfaction in SOEP and SHP, Controlling for Age

	SOEP (*n* = 3,811)			
	Level, *B* (*SE*)	Preretirement slope, *B* (*SE*)	Short-term slope, *B* (*SE*)	Postretirement slope, *B* (*SE*)
Intercept	6.31 (0.09)***	−0.07 (0.04)	0.10 (0.10)	−0.03 (0.02)
Retirement year (0 = 2001)	0.01 (0.00)	0.004 (0.00)**	0.02 (0.01)**	0.003 (0.00)*
Age	0.10 (0.01)***	0.00 (0.01)	−0.02 (0.01)	−0.01 (0.01)
Gender (0 = female, 1 = male)	−0.04 (0.04)	0.02 (0.01)	−0.05 (0.05)	0.03 (0.02)
Education (0 = low, 1 = middle and high)	0.41 (0.08)***	0.03 (0.03)	−0.03 (0.09)	0.02 (0.03)
Residual variance	1.96 (0.08)***	0.05 (0.01)***	0.42 (0.12)***	0.06 (0.01)***

	SHP (*n* = 1,806)			
	Level, *B* (*SE*)	Preretirement slope, *B* (*SE*)	Short-term slope, *B* (*SE*)	Postretirement slope, *B* (*SE*)
Intercept	7.29 (0.10)***	−0.09 (0.04)*	0.27 (0.13)*	−0.06 (0.04)
Retirement year (0 = 2001)	0.01 (0.00)	0.004 (0.00)**	−0.01 (0.01)	0.003 (0.00)*
Age	0.10 (0.01)***	0.00 (0.01)	−0.02 (0.01)	−0.01 (0.01)
Gender (0 = female, 1 = male)	−0.04 (0.04)	0.02 (0.01)	−0.05 (0.05)	0.03 (0.02)
Education (0 = low, 1 = middle and high)	0.41 (0.08)***	0.03 (0.03)	−0.03 (0.09)	0.02 (0.03)
Residual variance	1.36 (0.10)***	0.04 (0.01)**	0.12 (0.14)	0.04 (0.02)*

*Notes*: CFI = comparative fit index; CI = confidence interval; RMSEA = root-mean-square error of approximation; SHP = Swiss Household Panel; SOEP = German Socioeconomic Panel Study; SRMR = standardized root-mean-square residual; TLI = Tucker–Lewis index. Model fit: CFI = 0.994, TLI = 0.993, RMSEA = 0.017, 90% CI (0.013; 0.021); SRMR = 0.020.

*p < .05 ** *p* < .01 ***p < .001.

## Discussion

The macro-level conditions of retirement have changed over the last few decades, which may affect how an individual experiences retirement (Henning, Johansson, et al., 2022; Henning, Segel-Karpas, et al., 2022). In the present study, we investigated historical differences as well as cross-country differences in life satisfaction across retirement between 2000 and 2019 in Germany and Switzerland.

### Cross-Country Differences

Swiss retirees had a higher level of life satisfaction than German retirees before retirement, but in line with earlier studies, German retirees experienced a significant improvement in life satisfaction directly after the retirement transition (Wetzel et al., 2016). The lack of short-term effects among Swiss retirees is somewhat puzzling. However, it has been shown that short-term increases in life satisfaction in Switzerland depend on gender and postretirement behavior (Wernli et al., 2015). Thus, our analysis may have been too undifferentiated to find these effects.

The higher level of life satisfaction among Swiss participants is in line with the international ranking of Switzerland among the countries with the most satisfied citizens in the world (Helliwell et al., 2020). The fact that there were differences in levels of life satisfaction and short-time change, but not in pre- and postretirement development, reveals the advantages of considering retirement adjustment as a long-term multiphase process (Atchley, 1976) and separately investigating predictors of adjustment in different phases.

### Historical Differences in Level of and Change in Life Satisfaction

Our results showed that those who retired later in historical time exhibited higher levels and more positive changes in life satisfaction before retirement. These findings are in line with previous studies showing historically improved well-being in older age (Gerstorf et al., 2015) and higher retirement satisfaction (Henning, Johansson, et al., 2022) in Germany. Potential explanations for the positive development include historical improvements in different aspects of psychosocial and physical health (Hülür et al., 2016; Spuling et al., 2019), but also higher education and differences in the type of transition over time. However, a recent study did not find historical differences in changes across retirement between 1996 and 2014 in Germany (Henning, Segel-Karpas, et al., 2022). In the present study, the historical effects remained significant after controlling for gender and educational differences in the samples over time. However, when controlling for retirement age, the historical differences in levels of life satisfaction were no longer significant. There are several possible explanations for this: Retirement transitions have begun to occur at older ages, and if life satisfaction typically increases in one’s 60s anyhow (McAdams et al., 2012), those retiring at an older age should be more satisfied. Alternatively, there may be historical improvements in resources (e.g., health) that allow people to retire at older ages today but also be more satisfied.

Notably, the historical differences in change before retirement remained when controlling for age, and the effect of changes after retirement became significant as well. One possible explanation for the latter finding is that postretirement life has become more positive with historical time, but transitions happen at older ages when health declines, which affect life satisfaction, are more frequent. These effects may outweigh each other.

We expected that the early transition phase may have become less positive with historical time, given increased uncertainties in navigating retirement transitions (Hofäcker et al., 2015). Nevertheless, in contrast to hypothesis 1, we found historical improvements in short-term change in life satisfaction across retirement in Germany, although we had expected more negative developments due to the more substantial pension reforms implemented (e.g., an increase in the statutory retirement age from 65 to 67). The reasons for these improvements are, as yet, unknown. One potential explanation may be the historical differences in important resources (Henning, Johansson, et al., 2022; Wang et al., 2011). Higher levels of psychosocial resources are commonly associated with better short-term changes in well-being (Hansson et al., 2017). Alternatively, as global views on aging have become more positive in Germany (Beyer et al., 2017), the social status of being a retiree may have improved as well. Wetzel et al. (2016) saw short-term change when entering retirement as mainly influenced by a change in social status, and this change may have become more positive with historical time.

### Gender and Social Inequalities

The positive historical development in short-term change in Germany was not moderated by education, in contrast to Hypothesis 3 and in contrast to findings by Henning, Johansson, et al. (2022), who found historically improved retirement satisfaction to be only among former white-collar workers. It has been warned that pension reforms in the last decade have been disadvantageous for lower-educated workers (Hofäcker & Radl, 2016). However, these reforms were not associated with decreased levels of life satisfaction in this study. Nevertheless, pension reforms may affect future cohorts of retirees more strongly and consequences for life satisfaction in retirement may manifest in the next years and decades. Furthermore, we defined retirement as receiving pensions. If, instead, we would have defined it as a permanent exit from work, which can happen several years earlier or later, we may have observed stronger changes over historical time (cf. Turek et al., 2022).

We also expected that the increasing rates of late-life work participation among women and the changing gender roles would lead to stronger historical differences in the retirement transition among women than among men (Hypothesis 4). However, gender did not moderate the historical effects. The advantages and disadvantages for women may have possibly outweighed each other, or the effects of the historical developments are not yet observable but may become more pronounced in the next decades.

### Implications

As this is only one of a few studies on historical differences in well-being across retirement, and our design does not allow for causal inference, we are cautious about formulating political implications. Moreover, our study did not provide empirical evidence that the increasing complexity of retirement transitions and potentially rising social inequalities in retirement patterns negatively affect life satisfaction, at least not in the historical period and in the two countries we investigated. However, our results cannot be taken as evidence of the harmlessness of pension reforms either. Life satisfaction is an indicator that is well suited for the identification of the quality of the retirement adjustment process; however, it is also a subjective evaluation of the individual’s life circumstances, which are contrasted to the living situations of others. Thus, if the living situations of entire cohorts change, the individual’s evaluation might still remain unchanged. Consequently, there could be other indicators of retirement adjustment—such as health—that are more strongly affected by historical changes.

### Strengths and Limitations

The present study is one of the first to study the role of the historical context for individual adjustment to retirement (Henning, Johansson, et al., 2022; Henning, Segel-Karpas, et al., 2022), and, to our knowledge, the first to include Swiss data. A further contribution to the literature is the inclusion of a larger time span than previous studies and the distinction of preretirement change, level, and postretirement short- and long-term change in life satisfaction. The investigation of the potential moderation effects of gender and education further informs our knowledge of social inequalities.

Nevertheless, the present study also has several limitations. Because of the limitations of the data sets, as well as the complex methods and the large number of analyses, we have not scrutinized potential mechanisms for the historical differences. Future studies may determine whether historical differences in psychosocial resources (Hülür et al., 2016) can explain historical differences in life satisfaction across retirement.

Second, we have not considered the historically increasing diversity of retirement transitions. Our analyses did not include the type of transition (e.g. work → retirement vs nonwork → retirement, part-time vs full-time retirement), and we defined retirement transitions as pension reception. The rationale behind this approach is, on the one hand, that benefits facilitate retirement and, on the other hand, that eligibility ages affect normative expectations about what constitutes appropriate retirement age (National Academies of Sciences, Engineering, and Medicine, 2022). However, historical effects may differ by retirement definition and might have been more pronounced if subjective definitions of retirement had been the empirical focus. For instance, if individuals consider themselves retired when they stop working, the historical effects could have been more negative if more people received unemployment benefits before their “official” retirement. Nevertheless, as pointed out before, we consider our definition of retirement for reasons of comparability to be the most appropriate for the current study.

A further limitation is that we focused on mean-level differences and a few mainly contextual predictors instead of including various work–life factors, health, and psychosocial predictors of life satisfaction, as in previous studies (Henning et al., 2016). There seem to be subgroups of retirees with similar life satisfaction trajectories (Pinquart & Schindler, 2007), and future work may test if these subgroups and their characteristics have stayed the same with historical time or if new risk groups have evolved.

Finally, one potential shortcoming is representativeness. Although we excluded particularly unrepresentative subsamples in our analyses, our analyses may not be representative of the whole population and the whole time period. Some groups were oversampled in the survey and missing observations were most likely not at random. Nevertheless, we see no theoretical reason why missing patterns and unrepresentativeness should differ systematically over time or between surveys. Future research may use multicohort studies, which would allow the comparison of the trajectories of life satisfaction in specific birth cohorts (cf., Thorvaldsson et al., 2017) and which may also allow for a better inspection of representativeness for the whole population.

## Conclusion

Overall, our study showed some evidence for historical improvements in life satisfaction around the retirement transition in Germany and Switzerland, although short-term change has only shifted in Germany, and postretirement change seems to have remained the same. It is unclear yet if these average-level improvements hold for all parts of society and whether they will be long-lasting. More research is needed to investigate the potential reasons for historical differences in the experience of the retirement transition.

## Supplementary Material

gbad066_suppl_Supplementary_Materials

## Data Availability

The German Socio-Economic Panel Study (SOEP) data are available from SOEP due to third-party restrictions (for requests, please contact soepmail@diw.de). The scientific use file of the SOEP with anonymous microdata is made available free of charge to universities and research institutes for research and teaching purposes. The direct use of SOEP data is subject to the strict provisions of German data protection law. Therefore, signing a data distribution contract is a precondition for working with SOEP data. The data distribution contract can be requested with a form, available at: http://www.diw.de/soepforms. For further information, contact the SOEP hotline at either soepmail@diw.de or +49-30-89789-292. To access SHP data, please follow the instructions on this website: https://forscenter.ch/projects/swiss-household-panel/data/.

## Author Notes

1. https://osf.io/b7a9m/

2. https://osf.io/b7a9m/

3. An alternative model without a short-term effect and with pre- and postretirement slope set equal within each country had a significantly worse fit (Δ*χ*²(12) = 73.58, *p* < .001).
