# Supplementary material for: Historical and Cross-Country Differences in Life Satisfaction Across Retirement in Germany and Switzerland From 2000 to 2019
Source: J Gerontol B Psychol Sci Soc Sci. 2023 Jun 9;78(8):1365–74. doi: 10.1093/geronb/gbad066 (PMC10394993; doi:10.1093/geronb/gbad066)

**Supplementary Materials**

**Appendix A. Tables**

*Table S1.* Observations per wave and dataset

| **Dataset** | **LS-3** | **LS-2** | **LS-1** | **LS0** | **LS+1** | **LS+2** | **LS+3** | **LS+4** |
| --- | --- | --- | --- | --- | --- | --- | --- | --- |
| SOEP | 2,907 | 3,263 | 3,574 | 3,751 | 3,635 | 3,204 | 2,857 | 2,554 |
| SHP | 1,406 | 1,566 | 1,806 | 1,804 | 1,502 | 1,280 | 1,102 | 906 |

*Table S2.* Transitions per year and dataset

|  | SOEP | SHP |
| --- | --- | --- |
| 2001 | 213 | 51 |
| 2002 | 277 | 34 |
| 2003 | 246 | 47 |
| 2004 | 199 | 37 |
| 2005 | 198 | 60 |
| 2006 | 219 | 60 |
| 2007 | 199 | 80 |
| 2008 | 169 | 97 |
| 2009 | 194 | 89 |
| 2010 | 142 | 88 |
| 2011 | 188 | 99 |
| 2012 | 209 | 110 |
| 2013 | 180 | 100 |
| 2014 | 245 | 88 |
| 2015 | 235 | 158 |
| 2016 | 165 | 169 |
| 2017 | 188 | 138 |
| 2018 | 177 | 152 |
| 2019 | 168 | 149 |

*Table S3.* Unconditional piecewise growth curve model with equality constraints

|  | SOEP (*n* = 3,811) | | SHP (*n* = 1,806) | |
| --- | --- | --- | --- | --- |
|  | Mean (SE) | Variance (SE) | Mean (SE) | Variance (SE) |
| Level Life Satisfaction | 7.02 (0.03)*** | 2.01 (0.08)*** | 8.16 (0.03)*** | 1.39 (0.13)*** |
| Pre-Retirement Slope | 0.01 (0.01) | 0.05 (0.01)*** | 0.01 (0.01) | 0.10 (0.04)** |
| Short-Term Slope | 0.14 (0.03)*** | 0.43 (0.12)*** | -0.01 (0.03) | 0.12 (0.14) |
| Post-Retirement Slope | -0.01 (0.01) | 0.05 (0.01)*** | -0.01 (0.01) | 0.04 (0.02)* |

**p* < .05 ***p* < .01 ****p* < .001. Model fit: CFI = 0.994, TLI = 0.992, SMR = 0.024, RMSEA = 0.022, 90%CI[0.017;0.028].

*Table S4.* Predicting level and change in life satisfaction by retirement year (with equality constraints)

|  | SOEP (*n* = 3,811) | | | | SHP (*n* = 1,806) | | | |
| --- | --- | --- | --- | --- | --- | --- | --- | --- |
|  | Level  B (SE) | Pre-Retirement Slope  B (SE) | Short-Term  Slope  B (SE) | Post-Retirement Slope  B (SE) | Level  B (SE) | Pre-Retirement Slope  B (SE) | Short-Term  Slope  B (SE) | Post-Retirement Slope  B (SE) |
| Intercept | 6.87 (0.05)*** | -0.03 (0.02)* | 0.00 (0.05) | -0.02 (0.02) | 7.95 (0.06)*** | -0.05 (0.02) | 0.14 (0.08) | -0.05 (0.02)* |
| Retirement year (0 = 2001) | 0.02 (0.00)*** | 0.004 (0.00)*** | 0.01 (0.01)** | 0.00 (0.00) | 0.02 (0.00)*** | 0.004 (0.00)*** | - 0.01 (0.01) | 0.00 (0.00) |
| Residual Variance | 2.00 (0.08)*** | 0.05 (0.01)*** | 0.43 (0.12)*** | 0.06 (0.01)*** | 1.39 (0.10)*** | 0.04 (0.01)** | 0.12 (0.14) | 0.04 (0.02)* |

**p*< .05 ***p*< .01 ****p*< .001. Model fit: CFI = 0.994, TLI = 0.992, SRMR = 0.024, RMSEA = 0.021, 90%CI[0.016;0.026]

*Table S5.* Predicting level and change in life satisfaction in SOEP and SHP (without controlling for age at retirement, with interaction effects)

|  | SOEP (*n* = 3,811) | | | |
| --- | --- | --- | --- | --- |
|  | Level  B (SE) | Pre-Retirement Slope  B (SE) | Short-Term Slope  B (SE) | Post-Retirement Slope  B (SE) |
| Intercept | 6.62 (0.13)* | 0.00 (0.06) | 0.22 (0.14) | -0.11 (0.05)* |
| Retirement year (0 = 2001) | 0.02 (0.01) | 0.00 (0.01) | -0.02 (0.02) | 0.01 (0.01) |
| Gender  (0 = female, 1 = male) | 0.08 (0.09) | 0.05 (0.03) | -0.17 (0.09) | 0.06 (0.03) |
| Education  (0 = low, 1 = middle and high) | 0.25 (0.14) | -0. 07 (0.06) | -0.14 (0.15) | 0.07 (0.05) |
| Retirement year x Gender | -0.01 (0.01) | 0.00 (0.00) | 0.01 (0.01) | -0.01 (0.00) |
| Retirement year x Education | 0.00 (0.02) | 0.01 (0.01) | 0.03 (0.02) | -0.01 (0.01) |
| Residual Variance | 1.99 (0.08)*** | 0.05 (0.01)*** | 0.43 (0.12)*** | 0.06 (0.01)*** |
|  | SHP (*n* = 1,806) | | | |
|  | Level  B (SE) | Pre-Retirement Slope  B (SE) | Short-Term Slope  B (SE) | Post-Retirement Slope  B (SE) |
| Intercept | 7.34 (0.19)*** | -0.02 (0.06) | 0.31 (0.15)* | -0.14 (0.05)** |
| Retirement year (0 = 2001) | 0.02 (0.01) | 0.00 (0.01) | -0.02 (0.02) | 0.01 (0.01) |
| Gender  (0 = female, 1 = male) | 0.08 (0.09) | 0.05 (0.03) | -0.17 (0.09) | 0.06 (0.03) |
| Education  (0 = low,  1 = middle and high) | 0.64 (0.19)*** | -0. 07 (0.06) | -0.14 (0.15) | 0.07 (0.05) |
| Retirement year x Gender | -0.01 (0.01) | 0.00 (0.00) | 0.01 (0.01) | -0.01 (0.00) |
| Retirement year x Education | 0.00 (0.02) | 0.01 (0.01) | 0.01 (0.02) | -0.01 (0.01) |
| Residual Variance | 1.36 (0.10)*** | 0.04 (0.01)** | 0.11 (0.14) | 0.04 (0.02)* |

**p* < .05 ** *p* < .01 ****p* < .001. Model fit: CFI = 0.994, TLI = 0.993, RMSEA = 0.016, 90%CI[0.012;0.020]; SRMR = 0.022

*Table S6.* Predicting level and change in life satisfaction in SOEP and SHP, controlling for age,with interaction effects

|  |  |  |  |  |
| --- | --- | --- | --- | --- |
|  | SOEP (n = 3,811) | | | |
|  | Level  B (SE) | Pre-Retirement Slope  B (SE) | Short-Term Slope  B (SE) | Post-Retirement Slope  B (SE) |
| Intercept | 6.34 (0.13)*** | -0.01 (0.06) | 0.25 (0.14) | -0.09 (0.05) |
| Retirement year (0 = 2001) | 0.00 (0.01) | 0.00 (0.01) | 0.00 (0.02) | 0.01 (0.00) |
| Age | 0.10 (0.01)*** | 0.00 (0.01) | -0.01 (0.01) | -0.01 (0.00) |
| Gender  (0 = female, 1 = male) | 0.03 (0.09) | 0.05 (0.03) | -0.15 (0.09) | 0.03 (0.02) |
| Education  (0 = low,  1 = middle and high) | 0.34 (0.14)* | -0.07 (0.05) | -0.14 (0.15) | 0.06 (0.03) |
| Retirement year x Gender | -0.01 (0.01) | 0.01 (0.01) | 0.01 (0.01) | 0.00 (0.00) |
| Retirement year x Education | 0.01 (0.01) | 0.02 (0.02) | 0.02 (0.02) | 0.00 (0.01) |
| Residual Variance | 1.96 (0.08)*** | 0.05 (0.01)*** | 0.42 (0.12)** | 0.06 (0.01)*** |
|  | SHP (n = 1,806) | | | |
|  | Level  B (SE) | Pre-Retirement Slope  B (SE) | Short-Term Slope  B (SE) | Post-Retirement Slope  B (SE) |
| Intercept | 7.32 (0.14)*** | -0.02 (0.06) | 0.42 (0.16)* | -0.12 (0.05)* |
| Retirement year (0 = 2001) | 0.00 (0.01) | 0.00 (0.01) | -0.03 (0.02) | 0.01 (0.01) |
| Age | 0.10 (0.01)*** | 0.00 (0.01) | -0.01 (0.01) | -0.01 (0.01) |
| Gender  (0 = female, 1 = male) | 0.03 (0.09) | 0.05 (0.03) | -0.15 (0.09) | 0.06 (0.03)* |
| Education  (0 = low,  1 = middle and high) | 0.34 (0.14)* | -0.07 (0.05) | -0.14 (0.15) | 0.07 (0.05) |
| Retirement year x Gender | -0.01 (0.01) | 0.01 (0.01) | 0.01 (0.01) | -0.01 (0.00) |
| Retirement year x Education | 0.01 (0.01) | 0.02 (0.02) | 0.02 (0.02) | -0.01 (0.01) |
| Residual Variance | 1.36 (0.10)*** | 0.04 (0.01)*** | 0.12 (0.14) | 0.04 (0.02)* |
|  |  |  |  |  |

**p* < .05 ** *p* < .01 ****p* < .001. Model fit: CFI = 0.996, TLI = 0.995, RMSEA = 0.012, 90%CI[0.008;0.016]; SRMR = 0.024.

**Appendix B. Sample Selection**

For both countries, we included everyone who retired between two consecutive waves (i.e., who were not retired at one wave, and were retired at the next one). There were *n* = 6,417 transitions in the SOEP and *n* = 2,460 transitions in the SHP.

We further only included those whose retirement age was in line with the retirement ages in Germany and Switzerland. As mentioned before, we focused on retirement transitions into the pension system. Pension income before the actual retirement age may rather refer to the reception of widow pensions, unemployment benefits or disability pensions, which are taken out until one is eligible for old-age pensions. In Germany, pension laws usually included and still include different exceptions for specific groups (e.g., individuals with severe disabilities, women, long-term unemployed) and it was not possible to adjust the age for each of these groups. Instead, we included retirement ages of 60 and older, as retirement with 60 years was under particular circumstances possible during the whole period. Although rules are less complex and changeable in Switzerland, nevertheless, rules differed by gender and birth cohort as well. As it is possible to start receiving occupational pensions (but not public pensions) from 58 on and the dataset did not allow to distinguish type of pension, we included 58 as the earliest possible retirement date for the Swiss sample. We excluded *n* = 1,324 too early retirement transitions in the SOEP and *n* = 347 in the SHP. The actual statutory retirement age in both countries was 65 (64 for women in Switzerland) for most of the studied period, but it was raised gradually over birth cohorts in Germany, so the youngest birth cohorts in our study had to work a few months longer to be eligible to receive complete retirement pensions. For both countries, we therefore excluded very late retirement (older than 67 in the year of retirement*, n* = 457 in the SOEP, *n* = 308 in the SHP).

Furthermore, the SOEP includes some subsamples, which were unrepresentative for the German population (for example, refugee samples and a high-income sample) which could distort the result and potential historical effects, as they were drawn in specific years (*n* = 825).

**Appendix C.** **χ²-Tests**

**Unconditional Model**

Setting intercepts to be equal in both countries resulted in a significant loss of fit (Δχ²(1) = 338.65, *p* < .001). Setting the pre-retirement slope (Δχ²(1) = 0.80, *p* = .371) or the post-retirement slope (Δχ²(1) = 2.51, *p* = .113) to be equal in both countries did not result in a significant loss of fit. Setting the short-term slope to be equal in both countries again resulted in a significant loss in fit (Δχ²(1) = 25.66, *p* < .001).

**Historical Effects**

After adding retirement year as a predictor of all parameters, we set the effects of retirement year on the intercept (Δχ²(1) = 1.98, *p* = .160), the pre-retirement slope (Δχ²(1) = 0.20, *p* = .655) and the post-retirement slope (Δχ²(1) = 0.17, *p* = .682) equal between countries without significant loss in fit. However, the effect on the short-term slope seemed to differ between countries (Δχ²(1) = 15.94, *p* < .001).

**Historical effects by gender and education**

After adding gender and education as predictors of intercept and slopes to the model, the effects of retirement year on level (Δχ²(1) = 2.16, *p* = .142), pre-retirement change (Δχ²(1) = 0.10, *p* = .750) and post-retirement change (Δχ²(1) = 0.14, *p* = .707), were set equal across samples without significant loss in fit, but not the effect on short-term change (Δχ²(1) = 16.02, *p* < .001). Effects of gender could not be set equal (Δχ²(4) = 11.16, *p* = .049), but when we allowed the effects on the intercept to be the different, the model fit did not differ significantly anymore (Δχ²(3) = 2.53, *p* = .639). Effects of education (Δχ²(4) = 4.97, *p* = .291) were set equal without loss in fit as well.

Next, we added gender x retirement year and education x retirement year interaction effects, releasing equality constraints for the main effects again. The main effects of retirement year (Δχ²(4) = 4.57, *p* = .334) and gender (Δχ²(4) = 7.76, *p* = .101) were set equal without significant loss in model fit. The main effects of education could not be set equal (Δχ²(4) = 11.38, *p =* .022). Releasing the main effect of education on level of life satisfaction led to a model that did not differ significantly from the one with free main effects (Δχ²(3) = 2.50, *p* = .474). The retirement year x gender interaction effects were set equal across countries without significant loss in fit (Δχ²(4) = 8.58, *p* = .072).

However, when setting retirement year x education interaction effects to be the same, the model fit decreased significantly (Δχ²(4) = 15.54, *p* < .001). When we released the equality constraint for the effect on short-term change, the fit did not differ significantly from a model with all retirement year x education interaction effects (Δχ²(3) = 2.04, *p* = .564).

**Analyses for the model with age included**

*χ² Tests for the model without interaction effects*

Setting the effects of age to be the same across countries did not lead to a significant decline in model fit (Δχ²(4) = 7.37, *p* =.118). Effects of gender (Δχ²(4) = 5.02, *p* = .285) and education (Δχ²(4) = 4.83, *p* = .305) were set equal across countries without loss in fit too. Effects of retirement year on level, pre-retirement change and post-retirement change were set equal without loss in fit as well (Δχ²(3) = 0.63, *p* = .890), but the effect on short-term change could not be set equal without a significant loss in fit (Δχ²(1) = 14.97, *p* < .001).

*χ² Tests for the model with interaction effects*

Effects of age could be set equal across countries without a significant decline in model fit (Δχ²(4) = 8.23, *p* = .083), as well as main effects of education (Δχ²(4) = 1.32, *p* = .858) and gender (Δχ²(4) = 3.70, *p* = .448). The main effects of retirement year could not be set equal without significant loss in fit (Δχ²(4) = 11.62, *p* = .020), Releasing the effect on short term change led to a model that did not differ significantly from the one with all main effects of retirement year set free (Δχ²(3) = 4.92, *p* = .178). Gender x retirement year interaction effects (Δχ²(4) = 4.10, *p* = .392) and education x retirement year interaction effects (Δχ²(4) = 0.89, *p* = .926) were set equal without significant loss in fit.

*Table S5.*

*Figure S1.* Change in life satisfaction across retirement among SOEP and SHP participants. Samples differ significantly in pre-retirement level and short-term slope, but not in pre-retirement and post-retirement slope.


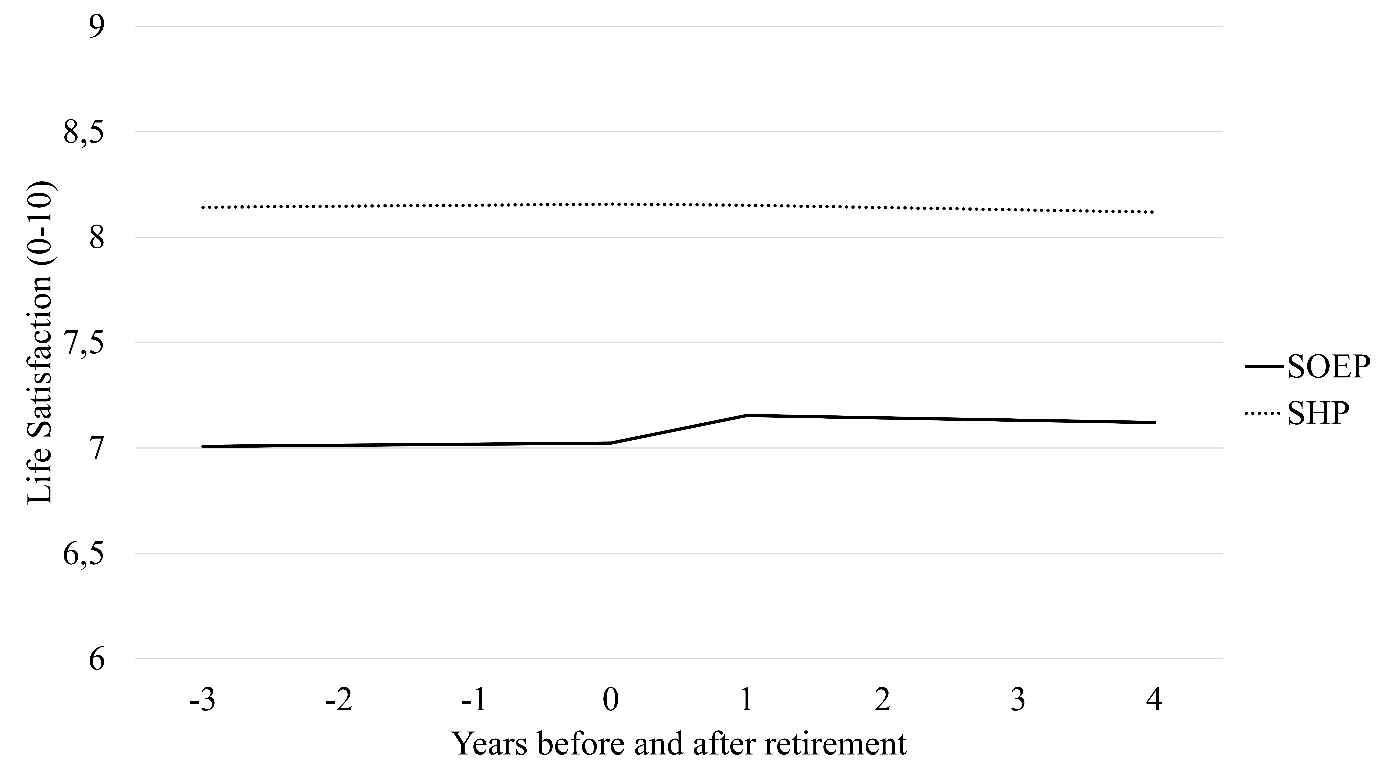

Supplement: gbad066_suppl_Supplementary_Materials [file gbad066_suppl_supplementary_materials.docx]
